# Kyste colloïde obstructif du troisième ventricule

**DOI:** 10.11604/pamj.2015.20.217.6264

**Published:** 2015-03-10

**Authors:** Abdellah Taous, Abdelhadi Rouimi

**Affiliations:** 1 Service de Neurologie, Hôpital Militaire Moulay Ismaïl, Mèknes, Maroc

**Keywords:** Kyste colloïde, troisième ventricule, hypertension intracrânienne paroxystique, colloid cyst, third ventricle, Paroxysmal intracranial hypertension

## Image en medicine

Il s'agit d'une patiente de 17 ans qui se plaignait depuis un mois d'une hémicrânie gauche positionnelle paroxystique, rebelle aux antalgiques simples, associée à des nausées, des vomissements et une diplopie d'instalation brutale apparue 48 heure avant son hospitalisation. L'examen Clinique a trouvé une paralysie de la 6^ème^ paire crânienne gauche et le fond d'œil a objectivé un œdème papillaire bilatéral stade III. Le bilan biologique était sans particularités. Une imagerie par résonance magnétique cérébrale (IRM) a montré une hydrocéphalie aigue obstructive biventriculaire et un processus lésionnel arrondi bien limité suspendu à la partie antéro-supérieure du troisième ventricule (V3), directement en arrière des trous de Monro, correspondant à un kyste colloïde. La patiente fut alors orientée en urgence en neuro-chirurgie pour prise en charge. Le kyste colloïde du V3 est une tumeur bénigne rare (0,5 à 2% des tumeurs cérébrales), naissant du neuro-épithélium primitif de la toile choroïdienne et survient habituellement entre 30 et 50 ans. Il siège toujours en arrière des trous de Monro, où il est appendu à la paroi antéro-supérieure du V3. Son éventualité doit être évoquée chez les patients présentant typiquement des poussées d'hypertension intracrânienne paroxystique (par un mécanisme de clapet) rebelles au traitement symptomatique. Il est mieux exploré par IRM notamment en coupes coronales et sagittales. Quoique bénin, le kyste colloïde nécessite une exérèse chirurgicale rapide, d'autant plus s'il existe une hydrocéphalie, en raison du risque de mort subite, par obstruction brutale du trou de Monro.

**Figure 1:**
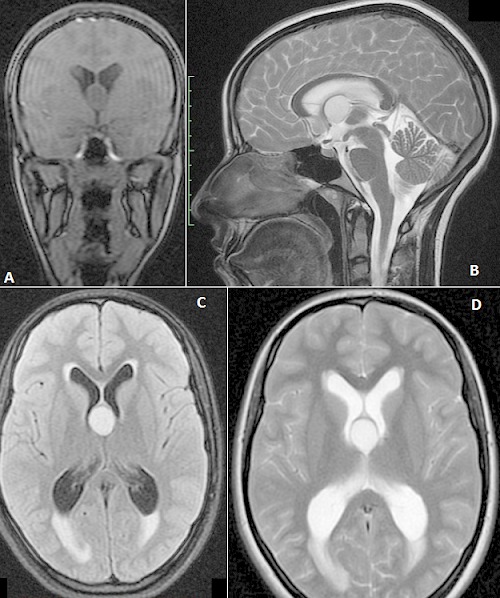
A) séquence T1 avec injection de Gadolinium en coupe coronale; B) séquence T2 en coupe sagittale; C) séquence Flair en coupe axiale; D) séquence T2 en coupe axiale

